# Nonsuperotemporal quadrant implantation of the Ahmed glaucoma valve using modified long scleral tunnel technique for intraocular pressure control

**DOI:** 10.5935/0004-2749.2022-0306

**Published:** 2024-02-23

**Authors:** Yasemin Un, Serhat Imamoglu

**Affiliations:** 1 Department of Ophthalmology, Istanbul Haydarpasa Numune Training and Research Hospital, Uskudar, Istanbul, Turkey

**Keywords:** Glaucoma/surgery, Sclera/surgery, Glaucoma drainage implant, Intraocular pressure, Tenon capsule

## Abstract

**Purpose:**

As superotemporal implantation of the Ahmed glaucoma valve is not always feasible in cases of refractory glaucoma, this study examined the characteristics and surgical outcomes of cases in which the valve was implanted in a nonsuperotemporal quadrant using a modified long scleral tunnel technique.

**Methods:**

This retrospective case-control study included 37 eyes with nonsuperotemporal quadrant--Ahmed glaucoma valve implantation in Group 1 and 69 eyes with superotemporal Ahmed glaucoma valve implantation in Group 2. The demographic characteristics of these groups, surgical outcomes, including complications, further surgical interventions, and surgical success rates were compared. Surgical success was defined as an intraocular pressure not exceeding 21 mmHg, accompanied by a minimum reduction of 20% in intraocular pressure from the baseline without any additional intraocular pressure-lowering procedures, and the absence of light perception loss or phthisis bulbi.

**Results:**

Group 1 had significantly higher numbers of eyes with secondary glaucoma and preoperative surgical procedures than Group 2 (p<0.05). Both groups had mean preoperative intraocular pressure values, and mean intraocular pressure values at the last visit of 34.2 and 27.9 months, 35.5 ± 1.5 and 35.8 ± 1.2 mmHg, and 14.5 ± 5 and 14.9 mmHg, respectively. Although both groups had 70.2% and 75.8% as their five-year cumulative probability of success, respectively, the rates of complications, revisional surgery, and additional surgical procedures did not differ significantly (p>0.05).

**Conclusion:**

The modified long scleral tunnel technique for Ahmed glaucoma valve implantation in nonsuperotemporal quadrants achieves intraocular pressure control and complication rates comparable to superotemporal implantation.

## INTRODUCTION

Ahmed glaucoma valve (AGV) implantation (New World Medical, Inc., Rancho Cucamonga, CA, USA) is a frequently performed surgical procedure for refractory glaucoma^(1,2)^. The routine implantation site is the superotemporal (ST) quadrant, where the surgical space between the lateral and superior rectus muscles is adequate and the eyelid protects the tube plate and covers most of the tube. However, in some cases where ST implantation is infeasible due to angle synechia, scarred conjunctiva, previous surgery, or scleral thinning^(3)^, non-ST quadrants are chosen for the implantation site.

AGV implantation can lead to various complications, with implant exposure via the conjunctiva being one of the most troublesome^(4)^. This can increase the risk of endophthalmitis and pose a threat to vision. To prevent such complications or tube-induced conjunctival erosion, several techniques have been described, including covering the tube with donor graft patches made of different biologic materials, preparing a long scleral tunnel to cover the tube, or using a short scleral tunnel with the Tenon duplication technique^(5-8)^.

Implanting AGVs in a quadrant other than the ST quadrant can be technically challenging. Therefore, this study investigated the surgical outcomes of non-ST quadrant AGV implantation using a modified long scleral tunnel technique.

## METHODS

We retrospectively reviewed patients who under-went AGV implantation for refractory glaucoma at the Ophthalmology Clinic of a single tertiary center between January 2015 and December 2021. The study followed the principles of the Declaration of Helsinki. The study sample was divided into two groups. Group 1 comprised 37 patients (37 eyes) who underwent AGV implantation in non-ST quadrants, including the superonasal (SN), inferonasal (IN), and inferotemporal (IT) quadrants, whereas Group 2 comprised 69 patients (69 eyes) who underwent AGV implantation in the ST quadrants.

The inclusion criteria for Group 1 comprised patients with refractory glaucoma of any age and etiology who underwent non-ST quadrant AGV implantation and had complete preoperative, surgical, and postoperative data available in their patient files, with a minimum follow-up of four months. Combined procedures were also included in Group 1. Group 2 included patients with refractory glaucoma of any etiology who underwent ST quadrant AGV implantation during the same period. Exclusion criteria included eyes with a previous history of glaucoma drainage device implantation, those with no light perception, and patients with less than four months of follow-up.

The same experienced glaucoma surgeon (S.I.) performed all surgical procedures using the modified long scleral tunnel method with a fornix-based approach, as described by Kugu et al.^(6)^. In all cases, AGV-FP7 (New World Medical, Rancho Cucamonga, CA, USA) and the same surgical technique were used, regardless of the implantation quadrant. The surgeon determined the implantation site based on the preoperative assessment of conjunctival motility, scar tissue, angle adhesions, and scleral appearance. If the ST quadrant was deemed unsuitable, the SN, IN, and IT quadrants were evaluated as possible implantation sites in order of preference.

The clinical data included several parameters, such as age, sex, best-corrected visual acuity (BCVA) measurements, Goldman applanation tonometry-based intrao-cular pressure (IOP) measurements, glaucoma medications, presence of diabetes mellitus (DM) and systemic hypertension (HT), type of glaucoma, cup-to-disk ratios, lens and corneal status, and previous ocular surgery. IOP changes, complications, additional interventions, BCVA, number of glaucoma medications, and success rates were investigated. Surgical success was defined as a minimum 20% reduction in IOP from the baseline, IOP not exceeding 21 mmHg, no need for implant removal or additional glaucoma surgery, absence of phthisis bulbi, and loss of light perception.

### Statistics

Statistical analyses were performed using the statistical package program IBM SPSS Statistics Standard Concurrent User V 26 (IBM Corp., Armonk, New York, USA). Numerical variables were compared between two groups using the t-test for independent samples if they followed a normal distribution or the Mann-Whitney U test if they did not. Linear mixed-model analysis was used to compare IOP and logMAR values, and the Bonferroni correction was applied to compare the main effects. The chi-square test was used to compare the categorical variables between the groups. Kaplan-Meier analysis was used to calculate the survival probabilities of the groups based on failure status, and log-rank (Mantel-Cox) analysis was utilized to compare the survival times between the groups. A p-value of less than 0.05 was considered statistically significant.

## RESULTS

The study included 106 eyes, with 37 eyes in Group 1 and 69 eyes in Group 2. The mean follow-up times for Groups 1 and 2 were 34.2 ± 27.2 and 27.9 ± 20.3 months, respectively (p>0.05). Among the eyes in Group 1, 23 (62%) had SN implantation, 11 (29%) had IN implantation, and 3 (8%) had IT implantation. Table 1 presents the demographic characteristics of the patients.

**Table 1:** Demographic Characteristics of the Groups

	Groups		Test Statistics	
	Group 1 n=37	Group 2 n=69	Test value	p-value
Sex, n (%)				
Male	26 (70.3)	45 (65.2)	0.278^†^	0.668
Female	11 (29.7)	24 (34.8)		
Age, (years)				
Mean ± SD	53.1 ± 17.1	57.8 ± 13.9	1.446^&^	0.148
M (min-max)	53 (20-85)	60 (13-81)		
DM, n (%)				
Absent	27 (73.0)	37 (53.6)	3.769^†^	0.063
Present	10 (27.0)	32 (46.4)		
HT, n (%)				
Absent	22 (59.5)	30 (43.5)	2.461^†^	0.154
Present	15 (40.5)	39 (56.5)		
BCVA, logMAR Mean ± SD	1.75 ± 0.90	1.51 ± 0.92	1.319^‡^	0.190
Cup-to-disk ratio Mean ± SD	0.90 ± 0.17	0.89 ± 0.18	0.299^‡^	0.766
Cornea, n (%)				
Graft with edema	2 (5.4)	1 (1.4)		
Clear graft	0 (0.0)	2 (2.9)		
Corneal opacity	1 (2.7)	1 (1.4)		
Corneal edema	13 (35.1)	13 (18.8)		
Clear cornea	21 (56.8)	52 (75.4)	6.362^†^	0.114
Lens, n (%)				
Aphakic	3 (8.1)	6 (8.7)	7.011^†^	0.061
Phakic	9 (24.3)	32 (46.4)		
Pseudophakic	22 (59.5)	30 (43.5)		
Scleral fixation IOL	3 (8.1)	1 (1.4)		
Glaucoma type, n (%)				
Aphakic	1 (2.7)	0 (0.0)	13.461^†^	**0.035**
Congenital glaucoma	2 (5.4)	1 (1.4)		
NVG	8 (21.6)	26 (37.7)		
POAG	5 (13.5)	9 (13.0)		
PACG	1 (2.7)	3 (4.3)		
PEXG	3 (8.1)	16 (23.2)		
Secondary	17 (45.9)^a^	14 (20.3)^b^		
Preop surgery, n				
Mean ± SD	1.5 ± 1.0	0.8 ± 0.6	3.857^&^	**<0.001**
M (min-max)	1 (1-5)	1 (0-3)		
Preop glaucoma medication, n				
Mean ± SD	3.5 ± 0.9	3.6 ± 0.5	0.203^&^	0.839
M (min-max)	4 (0-4)	4 (2-4)		
Follow-up				
Mean ± SD	34.2 ± 27.2	27.9 ± 20.3	0.395^&^	0.693
M (min-max)	25 (4-96)	20 (7-84)		

† = Chi-square test;

‡ = Independent-samples *t*-test;

& = Mann-Whitney *U* test, a= and b superscripts indicate differences between groups at secondary glaucoma type. Group 1= Non-ST implantation of the Ahmed glaucoma valve; Group 2= ST implantation of the Ahmed glaucoma valve. SD: standard deviation, M= median, DM= diabetes mellitus, HT= hypertension; BCVA= best-corrected visual acuity; IOL= intraocular lens; NVG= neovascular glaucoma; POAG= primary open angle glaucoma; PACG= primary angle closure glaucoma; PEXG= pseudoexfoliative glaucoma; Preop= preoperative.

**Figure 1:**
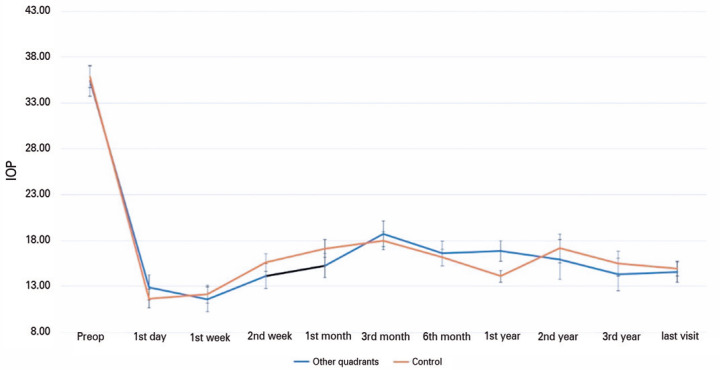
Mean intraocular pressures of Groups 1 and 2 before surgery and during the follow-up period. Error bars represent standard deviation.

Figure 1 presents the time-dependent IOP measurements of the groups. The mean preoperative IOP values of Groups 1 and 2 were 35.5 ± 1.5 and 35.8 ± 1.2 mmHg, respectively (p>0.05), and their mean IOP values at the last visit were 14.5 ± 5 and 14.9 mmHg, respectively. The mean decrease in IOP was 20.8 ± 1 (58.5%) mmHg in Group 1 and 20.9 ± 1 (58.3%) mmHg in Group 2 (p<0.001 for both).

Table 2 presents the early complications detected within the first postoperative month. The most commonly encountered complication was hyphema, which was observed in 8 (21.6%) eyes in Group 1 and 23 (33.3%) eyes in Group 2. This was followed by choroidal detachment in 6 (16.2%) eyes in Group 1 and 8 (11.6%) eyes in Group 2.

**Table 2:** Early Complications^*^

	Groups				Test Statistics	
	Group 1 n=37		Group 2 n=69		Test value^**^	p-value
	n	%	n	%		
Choroidal detachment	6	16.2	8	11.6	0.449	0.554
Hyphema	8	21.6	23	33.3	1.597	0.265
Endothelial touch	1	2.7	3	4.3	0.180	>0.999
Fibrinoid reaction	1	2.7	3	4.3	0.180	>0.999
Seidel test positivity	1	2.7	2	2.9	0.003	>0.999
Tube exposure	0	0.0	1	1.4	0.541	>0.999
Decompression retinopathy	0	0.0	1	1.4	0.541	>0.999
Narrow anterior chamber	1	2.7	3	4.3	0.180	>0.999
IOL subluxation	1	2.7	0	0.0	1.883	0.349
Corneal edema	0	0.0	2	2.9	1.093	0.541
Malignant glaucoma	0	0.0	1	1.4	0.541	>0.999
Suprachoroidal hemorrhage	1	2.7	2	2.9	0.003	>0.999
RD	0	0.0	1	1.4	0.541	>0.999
Wipeout	1	2.7	1	1.4	0.204	>0.999
None	17	45.9	23	33.3	1.631	0.215

* = Each complication was evaluated independently on a patient basis.

** Chi-square test. Group 1= Non-ST implantation of Ahmed glaucoma valve; Group 2= ST implantation of Ahmed glaucoma valve. IOL= intraocular lens; RD= retinal detachment.

Table 3 presents a comparison of the applied revisional procedures. The mostly encountered revisional procedure was needling with an antimetabolite, either mitomycin C, or 5-fluorouracil, applied at least once in 16 (43.2%) eyes in Group 1 and 30 (43.4%) eyes in Group 2 (p>0.05). The rates of conjunctiva-related revisional procedures and other procedures did not significantly differ between the groups (p>0.05).

**Table 3:** Comparison of Revisional Surgery Distributions Between the Groups^*^

	Groups				Test Statistics	
	Group 1 n=37		Group 2 n=69		Test value^*^	p-value
	n	%	n	%		
Needling number					1.518	0.904
One	11	29.7	16	23.2		
Two	4	10.8	11	15.9		
Three	1	2.7	2	2.9		
Four	0	0.0	1	1.4		
Fibrin glue application	1	2.7	1	1.4	0.204	>0.999
Amnion membrane suturing	1	2.7	1	1.4	0.204	>0.999
Tube tip shortening	4	10.8	5	7.2	3.801	0.120
Conjunctival auto-graft	0	0.0	1	1.4	0.541	>0.999
Conjunctival suturing	1	2.7	2	2.9	0.003	>0.999
Anterior chamber revision	1	2.7	5	7.2	2.814	0.160
Suprachoroidal hemorrhage drainage	0	0.0	1	1.4	0.541	>0.999
None	16	43.2	30	43.5	0.001	>0.999

* Each category was evaluated independently on a patient basis.

** Chi-square test. Group 1= Non-ST implantation of the Ahmed glaucoma valve; Group 2= ST implantation of the Ahmed glaucoma valve.

The rates of re-surgery did not significantly differ between the groups (Table 4). In one eye in Group 2, AGV expander implantation was performed due to a short tube tip, and AGVs were explanted in 2 (5.4%) eyes in Group 1 and 4 (5.8%) eyes in Group 2 due to tube exposure. Pericardial patch grafting was performed in 1 (2.7%) eye in Group 1.

**Table 4:** Comparison of Re-surgery and Additional Surgery Distributions Between the Groups

Re-surgery	Groups				Test Statistics	
	Group 1 n=37		Group 2 n=69		Test value^*^	p-value
	n	%	n	%		
None	27	73.0	50	72.5	6.101	0.833
AGV expander	0	0.0	1	1.4		
AGV explantation	2	5.4	4	5.8		
AGV tip repositioning	1	2.7	4	5.8		
Fibrosis and excision and AGV reimpl	0	0.0	1	1.4		
Fibrosis excision	1	2.7	3	4.3		
Pericardium patch grafting	1	2.7	0	0.0		
Additional surgery						
None	26	70.2	51	73.9	14.317	
PKP, phaco	1	2.7	1	1.4		
PPV	1	2.7	1	1.4		
DMEK	1	2.7	0	0.0		
DSAEK	1	2.7	0	0.0		
ECP	1	2.7	3	4.3		
TSCP	4	10.8	5	7.2		
Phaco	1	2.7	8	11.6		
IOL repositioning	1	2.7	0	0.0		

* Chi-square test. Group 1= Non-ST implantation of the Ahmed glaucoma valve, Group 2: ST implantation of the Ahmed glaucoma valve. reimpl= re-implantation; PKP= penetrating keratoplasty; phaco= phacoemulsification; PPV= pars plana vitrectomy, DMEK: Descemet’s membrane endothelial keratoplasty; DSAEK= Descemet’s stripping automated endothelial keratoplasty; ECP= endoscopic cyclophotocoagulation; TSCP= transscleral diode laser cyclophotocoagulation; IOL= intraocular lens.

Table 4 presents the distribution of additional surgical procedures. While 4 (10.8%) eyes in Group 1 and 5 (7.2%) eyes in Group 2 had transscleral diode laser cyclophotocoagulation, 1 (2.7%) eye in Group 1 and 3 (3%) eyes in Group 2 had endoscopic cyclophotocoagulation.

The mean number of glaucoma medications was 3.5 in Group 1 and 3.6 in Group 2 (p>0.05) preoperatively and decreased to 2.1 ± 1 and 2.2 ± 1, respectively, at the last visit (p>0.05), indicating a significant reduction for both groups (p<0.001).

The mean logMAR value was 1.7 for Group 1 and 1.5 for Group 2 (p>0.05) preoperatively and increased to 2 and 1.83, respectively, at the last visit (p>0.05). In both groups, the preoperative logMAR values increased significantly at the last visit (p>0.05 for Group 1 and p=0.001 for Group 2). The amount of increase in the logMAR values did not significantly differ between the groups (p>0.05).

The one-, two-, and five-year cumulative probability of success rates were 87.5%, 80.8%, and 70.2%, respectively, for Group 1, and 91.9%, 83.1%, and 74.8%, respectively, for Group 2. The survival times were 59.4 and 63.9 months for Groups 1 and 2, respectively (p>0.05).

**Figure 2:**
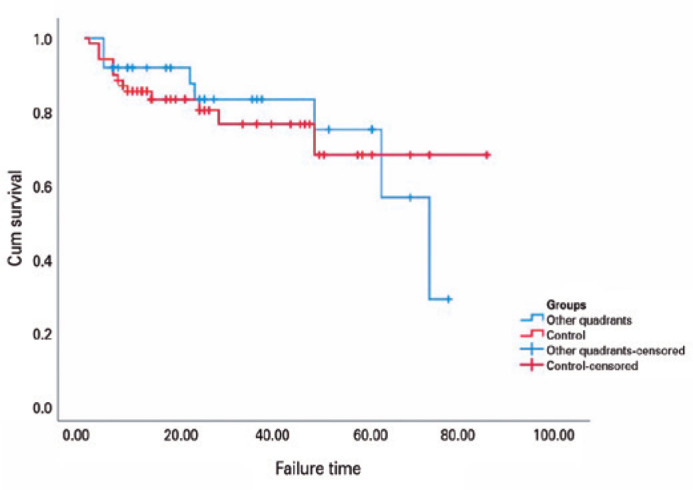
Kaplan-Meier survival probability curves of AGV as a function of time for Groups 1 and 2.

Figure 2 shows the Kaplan-Meier survival curves for both groups. In Group 1, surgical failure occurred in a total of eight eyes due to a lack of IOP control in six eyes and the surgical explantation of AGVs in two eyes. In Group 2, failure was observed due to a lack of IOP control in eight eyes, loss of light perception in two eyes, and surgical explantation of AGVs in four eyes.

**Figure 3:**
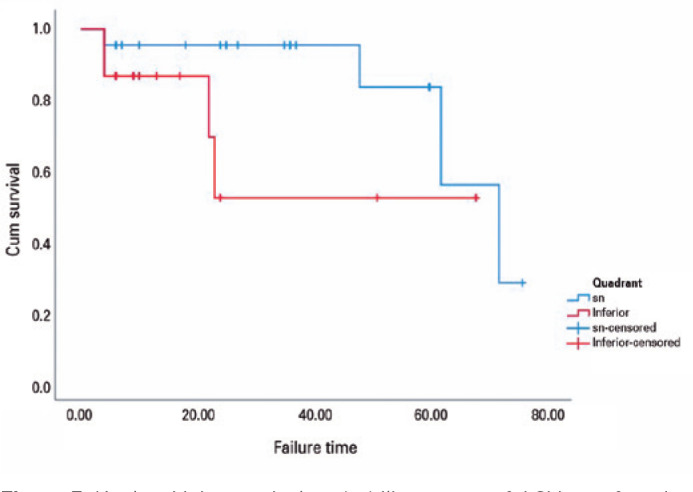
Kaplan-Meier survival probability curves of AGV as a function of time for superonasal insertions and inferior implantations.

In the subgroup analysis of Group 1, the preoperative mean of 32.7 ± 2 mmHg was reduced to a mean of 14.5 ± 1 mmHg in SN quadrant implantation, and the preoperative mean of 39.7 ± 3 mmHg was reduced to a mean of 14.4 ± 1 mmHg in inferior quadrant (IT and IN) implantations at the last visit (p<0.001 for both). The five-year cumulative probabilities of success were 83.5% and 52% for the SN and inferior quadrants, respectively. The survival times were 64.3 and 43.6 months for SN and inferior implantations (p>0.05). Figure 3 depicts the survival plots based on the implantation quadrant.

## DISCUSSION

In the current study, eyes undergoing AGV implantation in non-ST quadrants were found to undergo a higher number of preoperative surgical procedures and to have a higher rate of secondary glaucoma. Although the implantation of AGVs in non-ST quadrants is technically challenging, complications were similar to those of procedures performed in other quadrants. The five-year cumulative probabilities of success were found to be 74.7% and 67.8% in Groups 1 and 2, respectively, corroborating previously reported findings^(9,10)^. For example, Kang et al.^(11)^ found that the five-year cumulative probability of AGV implantation was 63.7% in 135 eyes. Topouzis et al.^(12)^ found a cumulative probability of success rate of 76% at 36 and 48 months after AGV implantation in a cohort of 60 eyes. Lee et al. determined that the cumulative success rate was 68.2% at 36 months in Korean patients^(13)^. In a multicenter study comparing AGV and the Baervalt shunt tube, the five-year cumulative failure rate of AGV implantation was reported to be 42% when an IOP of 6-21 mmHg was used as a criterion^(14)^.

The modified long scleral method employed in the current study was described by Kugu et al.^(6)^. This techni-que involves creating three scleral incisions at 10-12 mm, 6-8 mm, and 1.5-2 mm away from the limbus, which are then bonded together to create two consecutive scleral tunnels. Kugu et al.^(6)^ reported lower tube exposure rates with this method compared to the processed pericardium patch graft method, with exposure rates of 2.5% and 7.9%, respectively. In our study, we observed exposure in 3 (8%) patients in Group 1 and 4 (5.7%) patients in Group 2. Among the cases of exposure, further AGV explantation was performed on two patients in Group 1 and four in Group 2, and one eye in Group 1 was treated with donor scleral patch grafting. Our study demonstrated that the modified long scleral tunnel technique provided relatively safe coverage for the tube for both ST and non-ST implantations. The rates of re--surgery did not differ significantly between the groups.

Tamcelik et al.^(8)^ described a useful method with a low exposure rate for AGV implant surgery, which involves the creation of a short scleral tunnel with Tenon advancement and duplication. They reported, in a multicenter study comparing three different surgical techniques for the long term, an exposure rate of 12.9% for AGV implant surgery without patch grafts and 2.2% for AGV implant surgery with donor scleral patch grafts, while no exposure occurred in any of the cases in which the combined short scleral tunnel with Tenon advancement with duplication technique was used. The implantation site employed in most cases in the report was the superior hemisphere, and this technique appeared to provide very safe protection for the tube.

Several studies have compared the results of superior versus inferior AGV implantation. Pakravan et al.^(15)^ evaluated the results of 58 superior and 48 inferior implantations at 10 months and reported one-year success rates of 81.8% and 95.2%, respectively. Despite the success rate comparability, the overall rate of complications, such as exposure requiring implant removal, a cosmetically unappealing appearance, and endophthalmitis, was higher in the inferior group (12 eyes, 25%) than in the superior group (3 eyes, 5.2%).

Rachmiel et al.^(16)^ investigated the intermediate-term results of 31 eyes with superior implantation and 52 eyes with inferior implantation and found similar success rates (71.5% and 64.6%, respectively) at 36 months. The authors observed more complications in the inferior implantation group, but only wound dehiscence, and transient diplopia showed a statistically significant difference. Notably, our study did not compare superior and inferior implantations because most of our patients underwent ST and SN implantations.

Our study revealed comparable success rates between the SN quadrant and the IN and IT quadrants. The five-year cumulative probabilities of success were 83.5% and 52% for the SN quadrant and inferior quadrants, respectively. The survival times were 64.3 and 43.6 months for SN and inferior implantations, respectively (p>0.05). Although not statistically significant, superior implantation had a better survival rate. Failure occurred due to tube exposure and explantation in one patient, each in the SN, and inferior implantation groups. The remaining failed cases were due to a lack of IOP control. However, caution must be exercised when interpreting the similar complication rates between the SN and inferior cases because our cohort included only 23 SN and 14 inferior implantations. Therefore, the number of patients was insufficient for further comparisons.

In the current study, both Groups 1, and 2 showed a significant decrease in BCVA from the preoperative period to the last visit. The mean logMAR values for Groups 1 and 2 were 1.7 and 1.5, respectively, in the preoperative period, which increased to 2, and 1.83, respectively, at the last visit. In the Ahmed versus Baerveldt (AVB) study, the mean logMAR acuity in the Ahmed group deteriorated from 1.2 ± 1.0 preoperatively to 1.5 ± 1.2 at five years^(14)^. As the current cohort comprised eyes with worse visual acuity, their last-visit BCVA values were also worse than those reported in the AVB study. Because eyes requiring AGV implantation already have refractory glaucoma and a poor prognosis^(17)^, they are expected to have lower visual acuity due to not only glaucoma progression and primary pathologies but also complications related to AGV implantation, such as corneal pathologies, cataracts, hypotony maculopathy, and wipe-out syndrome^(18,19)^.

Groups 1 and 2 showed a significant decrease in the number of glaucoma medications from the preoperative period to the last visit. While the mean preoperative IOP values were 35.5 ± 1.5 and 35.8 ± 1.2 mmHg in Groups 1 and 2, respectively, these values decreased by 58.5% and 58.3% at the last visit. Preoperative and postoperative IOP levels did not significantly differ between the groups, except for the first year, where the mean IOP levels were significantly higher in Group 1, which is probably related to the hypertensive phase (HP). However, the number of needling procedures required did not differ significantly between the groups. In a recent report investigating the risk factors for HP, a higher preoperative IOP, and a younger age were found to be risk factors^(20)^. In the same report, HP, defined as an IOP rise within the postoperative three months, was mostly encountered in traumatic glaucoma cases. HP develops approximately four weeks after implantation and lasts at least 12 to 16 weeks^(21)^. While most eyes with HP do not show improved IOP control, they usually require the same number of glaucoma medications as the during HP period^(22,23)^.

Despite the retrospective design and low number of patients employed in this study, we were able to provide important results through the long-term comparison of ST and non-ST quadrant implantations. Most studies have mainly focused on comparing superior and inferior implantations, whereas our study stands out, as it presents the results of the comparison of ST and non-ST quadrant implantations, which is a notable aspect of our study.

In conclusion, we found that eyes that underwent more preoperative ocular procedures and those with secondary glaucoma required non-ST quadrant AGV implantation at a higher rate. We also found that non-ST quadrant AGV implantation using the modified long scleral tunnel method was as successful as ST quadrant implantation. Therefore, if ST AGV implantation is infeasible, non-ST quadrant implantation with the modified long scleral tunnel method is a reliable option. Furthermore, although statistical significance-based evidence was not provided, we infer that SN quadrant implantation may be a preferable option over inferior quadrant implantation. Overall, the rates of complications, and the need for further surgery were comparable between non-ST and ST implantations.
